# Mesoscale Model for Composite Laminates: Verification and Validation on Scaled Un-Notched Laminates

**DOI:** 10.3390/polym16121659

**Published:** 2024-06-11

**Authors:** Giuseppe Corrado, Albertino Arteiro, António Torres Marques, Fernass Daoud, Florian Glock

**Affiliations:** 1 DEMec, Faculdade de Engenharia, Universidade do Porto, Rua Dr. Roberto Frias, s/n, 4200-465 Porto, Portugal; 2 Stress Methods and Optimisation, Airbus Defence and Space GmbH, 85077 Manching, Germany; 3 Department of Aerospace and Geodesy, Technical University of Munich, 85521 Ottobrunn, Germany

**Keywords:** composites, failure, size effect, CFRP, damage model, finite element analysis (FEA)

## Abstract

This paper presents a mesoscale damage model for composite materials and its validation at the coupon level by predicting scaling effects in un-notched carbon-fiber reinforced polymer (CFRP) laminates. The proposed material model presents a revised longitudinal damage law that accounts for the effect of complex 3D stress states in the prediction of onset and broadening of longitudinal compressive failure mechanisms. To predict transverse failure mechanisms of unidirectional CFRPs, this model was then combined with a 3D frictional smeared crack model. The complete mesoscale damage model was implemented in ABAQUS^®^/Explicit. Intralaminar damage onset and propagation were predicted using solid elements, and in-situ properties were included using different material cards according to the position and effective thickness of the plies. Delamination was captured using cohesive elements. To validate the implemented damage model, the analysis of size effects in quasi-isotropic un-notched coupons under tensile and compressive loading was compared with the test data available in the literature. Two types of scaling were addressed: sublaminate-level scaling, obtained by the repetition of the sublaminate stacking sequence, and ply-level scaling, realized by changing the effective thickness of each ply block. Validation was successfully completed as the obtained results were in agreement with the experimental findings, having an acceptable deviation from the mean experimental values.

## 1. Introduction

In structural testing, the building block approach (BBA) is the main reference used for the certification of composite structures [1]. Following this approach, several tests are performed on small size specimens under different conditions and fewer when moving upward in the structural testing pyramid. The goal of this process is to gain a deep understanding of the structural behavior under simple loading conditions at the early stages of the design process, where any change in the design can still be made without incurring unfeasible costs. This approach is implemented for experimental campaigns as well as for virtual testing [2].

In this context, understanding and prediction of size effects (or scaling effects) in fiber-reinforced polymer (FPR) laminates has increasingly become an important area of research. Indeed, since the design of composite structures is mostly based on the mechanical behavior of small coupons (as supported by the BBA), understanding the effect of up-scaling on the strength and failure behavior of continuous FRP structures is fundamental.

A significant effort has been made to document and predict the scaling effect on un-notched (UNT) [3,4,5,6,7] and notched (NT) [8,9,10,11,12] specimens. For a detailed review of size effects in FRP laminates, including the size effects in smooth (UNT) coupons, hole size effects in tension and in compression, and the effects of laminate and ply thickness scaling, the reader is referred to Ref. [13].

Thanks to this comprehensive research work, important trends have been outlined, highlighting how the strength of composite laminates changes by applying specific geometrical scaling. Moreover, these studies have developed an understanding of the failure mechanisms controlling the strength of composite specimens of different dimensions. For instance, it has been experimentally demonstrated that fiber-dominated failure modes lead to higher UNT strengths and lower NT strengths as the structural dimensions increase, while the opposite has been observed for matrix-dominated failure modes, including delamination.

In particular, Wisnom et al. [4] found a significant size effect in IM7/8552 carbon-epoxy laminates: (i) the increase in tensile strength of quasi-isotropic (QI) specimens when the size was scaled up by repeating the sublaminate stacking sequence (“n-scaling”, [45/90/−45/0]_ns_) and (ii) the decrease in tensile strength when the specimen size was scaled up increasing the effective ply thickness (“m-scaling”, [45_m_/90_m_/−45_m_/0_m_]_s_). On the other hand, for QI UNT specimens under longitudinal compression, Lee and Soutis [5] presented the following results for the same material: no evidence of a size effect when the specimens are scaled up at the sublaminate level (i.e., with “n-scaling”) and the decrease in compressive strength of ply-level scaled (i.e., “m-scaled”) specimens.

Regarding critical failure mechanisms, experimental results on UNT specimens with sublaminate scaling under tension showed that all specimens exhibited no visible (superficial) damage prior to ultimate failure, with a clean break across the width in the gauge section. The smallest n = 1 specimen is the only case where final failure also involved delamination [4].

Unlike sublaminate-scaling, ply-level scaled specimens under tensile loading exhibited a different size effect behavior [4]. In the specimen with m = 2, superficial matrix cracks were observed in the range of 343–396 MPa, followed by delamination at the free edges that propagated until catastrophic fiber failure. A similar pattern in the initiation of failure took place for the specimens with m = 4, but matrix cracks in the 90° plies and delamination between the 90° and −45° plies were also observed. Final failure occurred as a combination of the complete delamination between the −45° and 0° plies and subsequent fiber failure. The sequence of failure events in the specimens with m = 8 and m = 4 is similar, with some initial transverse cracking observed on the surface [4].

The experimental findings for the compressive strength of QI UNT specimens showed no significant size effects with ply-level or sublaminate-level scaling [5]; for all cases, failure took place in the gauge length, driven by the compressive strength of the inner blocked 0° plies. However, the extensive matrix cracking that was introduced during the cutting process of the test pieces could contribute to the initiation of edge delamination and premature specimen failure [5]. These defects led to conservative results, especially in the 8 mm-thick IM7/8552 blocked laminate, as explained in Ref. [5]. Further, a tendency of failures near the grip for m ≥ 4 specimens was pointed out, which suggests that blocking the plies makes the laminate more susceptible to stress concentrations developed in the tab region.

Regarding the prediction of size effects in FRP laminates, while the prediction of scaling effects on NT coupons is a widely studied topic [14,15], the prediction of scaling effects and the governing failure modes in UNT coupons is still unexplored. Advanced failure criteria can be employed for “hot-spot” identification of the most critical areas for failure onset, but they are not able to predict the ultimate load of general multidirectional laminates since the failure of these materials is governed by a progressive evolution of damage [16]. Therefore, detailed damage models for composite materials are required to predict damage evolution up to collapse in FRP laminates by accounting for the complex interactions between intralaminar and interlaminar (delamination) damage mechanisms.

The progress in computational power has allowed the research community to perform deeper studies, considering a level of resolution that was not possible before. Exploiting these technological advancements, reliable numerical models representing the different types of damage mechanisms, with the respective damage evolution laws, have been developed at different mechanical scales. In fact, the ability of damage models to predict physical phenomena, such as the initiation and propagation of damage, strongly depends on the scale at which the damage mechanisms under consideration are modeled. Considering the smallest scale of composites idealization, the choice would be numerical modeling at the micro-mechanics scale. This would allow us to treat the constituent materials as homogeneous, considering fibers and matrices separately. However, the available models at this scale have a low degree of maturity and, at the same time, are computationally demanding [17,18,19,20,21,22,23,24,25]. Therefore, approaches at the micro-scale are still not considered suitable to model damage evolution up to the final collapse of composite laminates.

On the other hand, the multiscale nature of damage and failure in composite structures has been the motivation for recently proposed multiscale models. In multiscale modeling, macroscopic and microscopic models are coupled to take advantage of the efficiency of macroscopic models and the flexibility of microscopic models [26]. The scope of such multiscale modeling is to design combined macroscopic–microscopic computational methods that are more efficient than solving the full microscopic model. Multiscale models can be categorized into two groups: hierarchical and concurrent. In hierarchical models, homogenization is employed to transfer the predicted behavior of the material from a smaller scale (e.g., micromechanical representative volume element) to a larger scale. Models based on this approach have been implemented in commercial FE packages. On the other hand, in concurrent multiscale models, instead of applying homogenization to bridge the scales, both scales are concurrently present in the model. In undamaged areas, a macro-model with effective homogenized behavior is employed, while in damaged areas, the model resolution is increased and macro–micro features are explicitly accounted for [27]. Despite significant progress, a number of outstanding challenges have so far limited the impact of this approach on the composites community. First is the issue of computational complexity: multiscale modeling requires performing (coupled or uncoupled) simulations at multiple time and length scales. Significant computational resources are typically needed to predict the response using multiscale approaches. Second, multiscale models require calibration of model parameters that operate at multiple time and length scales [27].

So far, the meso-mechanics scale has been proven to be a successful trade-off, able to accurately represent the quasi-brittle behavior of laminated composite structures with a reasonable computational cost. At this scale, plies are the building blocks of the model, and the effects of delamination are usually treated separately from intralaminar damage mechanisms.

The typical procedure to predict intralaminar damage is as follows:

1. Lamina strain and stress analysis;
2. Application of lamina failure criteria;
3. Application of damage evolution models (as a function of the failure mode predicted at the lamina level).

Damage evolution (point three) is usually modeled by degrading the ply elastic properties to represent the occurrence of fiber breakage and matrix cracking [16,28]. This degradation may be sudden or progressive.

For transverse matrix cracking, the progressive degradation of elastic properties represents the progressive accumulation of transverse cracks until the crack density saturation is achieved. The reduction in transverse elastic properties can be a function of the stress state. This consideration is reasonable since a matrix crack under compressive stresses can still carry some load. For longitudinal damage mechanisms, the progressive degradation of elastic properties represents the accumulation of fiber breaks and subsequent pull-out in tension, as well as the onset and broadening of fiber kinking in compression.

Models at the meso-mechanical scale are typically divided into two different approaches: the continuum damage mechanics (CDM) approach and the discrete damage mechanics (DDM) approach. The framework of continuum damage mechanics, introduced for the first time by Kachanov [29] for the description of creep in metals, is a widespread approach for the description of the inelastic response of materials with damage. In general, the formulation of a CDM model starts with the definition of a potential (the complementary free energy density) as a function of damage variables associated with different failure modes. It is also necessary to define the damage activation functions, i.e., the conditions that lead to the onset of inelastic response and the damage evolution functions. A complete definition of a CDM model for the simulation of intralaminar damage can be found, for instance, in Refs. [30,31]. The CDM model simulates localized intralaminar damage using strain-softening constitutive models. To avoid mesh-dependent solutions, the energy dissipated is regularized for each damage mechanism using a modification of the crack band model [32]. Additionally, a maximum allowable element size is defined to avoid physically unacceptable snap-backs of the material response [31].

However, continuum approaches alone cannot realistically simulate the response of laminates dominated by interlaminar mechanisms, which are discrete in nature and sensitive to the local stress fields and boundary conditions. A more effective computational approach in such cases would have to include a concurrent combination of smeared and discrete models to capture the interaction between the material and structural (or local and global) behavior. For this reason, DDM can be a suitable approach to progressive failure modeling when delamination is explicitly introduced into the model via cohesive zone models to predict the displacement discontinuities that they create [27]. Alternatively, embedded discontinuity models, such as the extended finite element method (X-FEM), can be used to insert cracks (and delamination) into locations that are independent of the mesh orientation and without the need for re-meshing [33].

To predict the ultimate strength of composite structures, it is necessary to have an accurate numerical representation of all damage modes and their interactions. Some of the most complex damage modeling frameworks available rely on CDM to represent intralaminar damage modes (e.g., transverse matrix cracking and fiber failure) and cohesive zone models to capture delamination between ply interfaces.

The goal of this paper is to present a mesoscale damage model through rigorous verification and validation, including the prediction of scaling effects in UNT coupons and the associated damage mechanisms and failure modes. The methodology introduced in this work consists of a composite material model proposed in the literature [34], representing the quasi-brittle behavior of composite structures. To account for the effect of complex 3D stress states during the onset and broadening of longitudinal compressive failure mechanisms, a 3D invariant-based failure theory [35,36] was implemented not only for damage initiation [34], but also for damage propagation by finding the longitudinal component of the stress tensor at the intersection with the failure surface, and including it in the damage dissipation function. The formulation of the constitutive model implemented for this work is described in the next section, followed by the verification and validation of the model to predict scaling effects in UNT composite laminates.

## 2. Material Model

**Figure 1:**
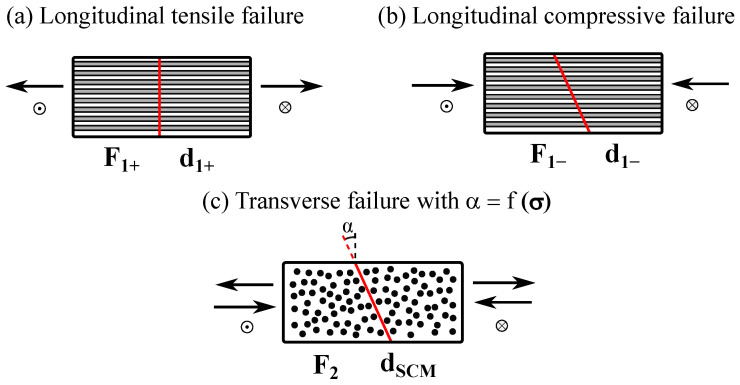
Fracture surfaces resulting from the different damage mechanisms and corresponding damage activation and propagation functions.

The constitutive model implemented for this work assumes that the mechanical behavior prior to damage initiation is linear–elastic. Then, for the definition of the damage model, suitable damage activation functions need to be formulated for each of the addressed failure modes. Herein, the meso-scale damage model aims at representing three damage mechanisms, schematically represented in Figure 1: longitudinal tensile fracture, longitudinal fiber kinking failure, and transverse matrix cracking with a fracture angle α, parallel to the fiber direction and dependent on the applied stress state (σ).

To account for the effect of complex 3D stress states, 3D invariant-based failure criteria [35,36] are implemented as damage activation functions. This set of criteria is coupled with a smeared crack model (SCM) for transverse cracking and continuum damage mechanics (CDM) models for fiber-dominated damage, which together account for the kinematics of matrix cracking and fiber tensile or compressive fracture during damage propagation. Furthermore, to predict delamination, cohesive elements are used at the interfaces between layers with different orientations.

**Figure 2:**
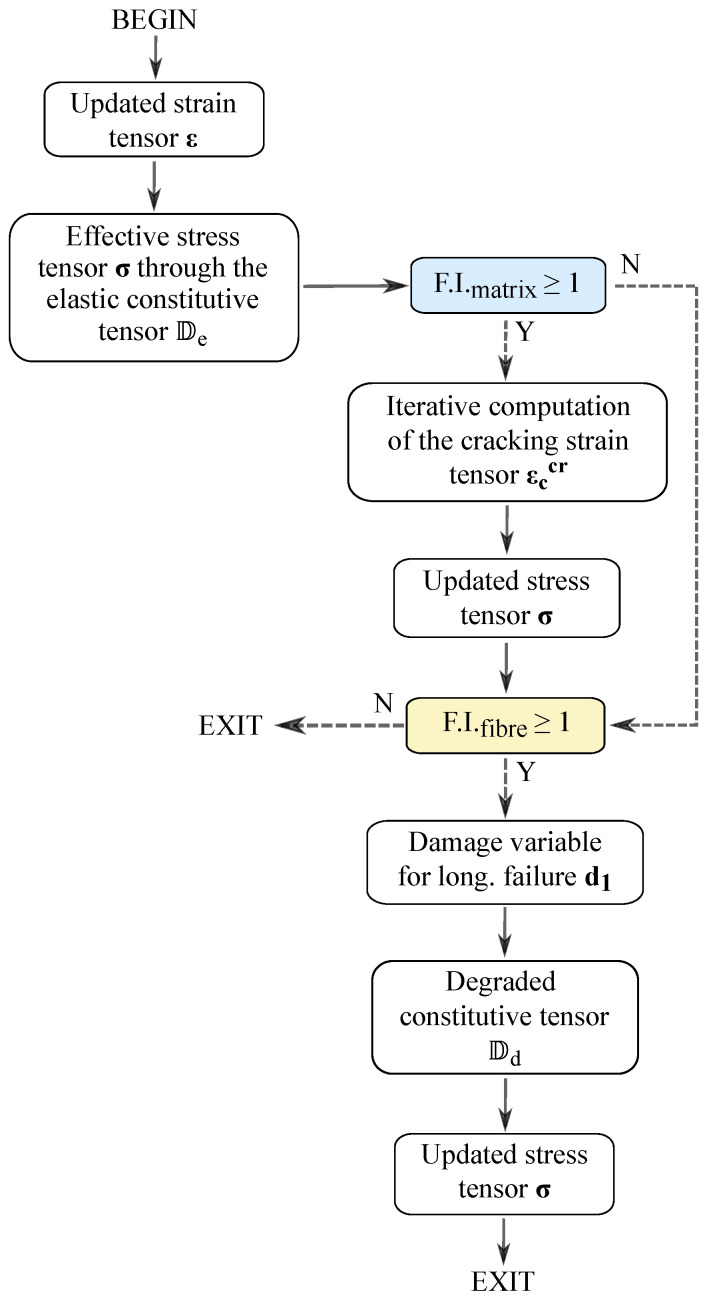
Flowchart of the meso-scale damage model.

This mesoscale damage model is implemented using the FE package ABAQUS^®^/Explicit 2020 [37], which is an explicit dynamics analysis solver based on the implementation of an explicit integration rule. The flowchart of the implemented model is presented in Figure 2, with the cracking strain tensor being computed iteratively (as presented in Ref. [38]), while the CDM model from Refs. [30,31] predicts the damage evolution for longitudinal failure mechanisms.

In the previous implementation of the CDM model presented in Refs. [34,39], the 3D invariant-based kinking model was implemented as a damage activation function, while the damage evolution law for longitudinal compressive failure was formulated using the maximum stress criterion. This represented the limitation of the CDM model for the description of fiber-dominated damage, and for this reason, a modification in the corresponding damage evolution law is proposed in this work to account for the effects of general 3D stress states. The following sections describe the mesoscale damage model, as illustrated in Figure 2, starting with the SCM and then presenting the CDM model, including the details of the latest developments.

### 2.1. Smeared Crack Model for Transverse Matrix Cracking

The onset of transverse failure was predicted using the 3D invariant-based failure criterion for matrix-dominated failure [35,36]. To ensure that transverse cracking initiates at the intersection with the failure surface, i.e., when the condition $f_{M}\left(\sigma \right)=1$ is met, an iterative scheme based on the Newton–Raphson method was employed, following Refs. [40,41].

Exploiting the accurate prediction of the stress state at the intersection with the failure surface, the fracture angle α is calculated by means of a pragmatic approach proposed in Refs. [34,35,39,41]. It is worth noting that the orientation of the matrix-dominated fracture plane is not known in advance, as it depends on the stress state triggering matrix cracking and on the material properties. Moreover, the present model assumes that the angle of the fracture plane α remains constant after damage initiation [38]. The pragmatic approach for the determination of the fracture angle α is summarized in Table 1.

**Figure 3:**
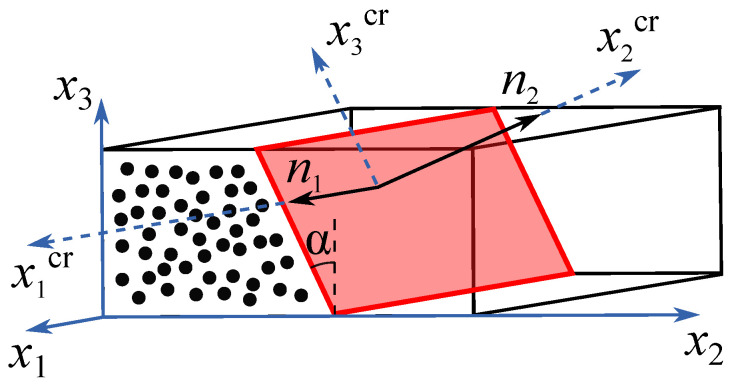
Transverse fracture plane, modeled using Ref. [38].

A schematic illustration of the transverse fracture plane is shown in Figure 3, where the coordinate system $x_{1}\;x_{2}\;x_{3}$ is aligned with the material axes and the $x_{1}^{cr}\;x_{2}^{cr}\;x_{3}^{cr}$ cracking coordinate system ($x_{1}^{cr}\;x_{2}^{cr}\;x_{3}^{cr}$) is obtained with a rotation of an angle α around the $x_{1}$ axis. Therefore, the unit vector normal to the fracture plane can be expressed as follows:

(1) $$n_{2}={\left\{\;0,\;\;\cos\alpha ,\;\;\sin\alpha \right\}}^{T}.$$

The SCM uses an additive decomposition of the strain tensor, which is divided into elastic strain $\varepsilon_{e}$ and cracking strain $\varepsilon_{c}$, and it is based on a cohesive law that relates the tractions acting on the fracture plane with the corresponding displacement jumps across the crack faces. Therefore, the definition of the strain tensor by additive decomposition can be expressed as follows:

(2) $$\varepsilon =\varepsilon_{e}+\varepsilon_{c}=\varepsilon_{e}+R\cdot \varepsilon_{c}^{cr}\cdot R^{T},$$

where $\varepsilon_{c}^{cr}$ is the cracking strain projected in the crack coordinate system, and R is the rotation matrix relating the global coordinate system associated with the fracture plane.

The stress–strain relation is given as follows [38]:

(3) $$\sigma =D_{e}\;:\;\left(\varepsilon -\varepsilon_{c}\right)=D_{e}\;:\;\varepsilon -D_{e}\;:\;\left(R\cdot \varepsilon_{c}^{cr}\cdot R^{T}\right).$$

Therefore, the tractions acting on the fracture plane, using Equation (3), can be expressed as follows [38]:

(4) $$t^{cr}=R^{T}\cdot \left(\sigma \cdot n_{2}\right)=R^{T}\cdot \left[D_{e}\;:\;\varepsilon -D_{e}\;:\;\left(R\cdot \varepsilon_{c}^{cr}\cdot R^{T}\right)\right]\cdot n_{2}.$$

Following Ref. [38], the tractions can be also obtained from the cohesive law of the cracking band with the displacement jump on the fracture plane ($\omega^{cr}$), where the latter is calculated as a function of the cracking strain tensor and the characteristic length of the finite element ($l^{*}$):

(5) $$\omega^{cr}=\left[\left(2\varepsilon_{c}^{cr}:n_{1}^{cr}⊗n_{2}^{cr}\right)n_{1}^{cr}+\left(\varepsilon_{c}^{cr}:n_{2}^{cr}⊗n_{2}^{cr}\right)n_{2}^{cr}+\left(2\varepsilon_{c}^{cr}:n_{2}^{cr}⊗n_{3}^{cr}\right)n_{3}^{cr}\right]\;l^{*}.$$

In the present implementation, $l^{*}$ is taken as the square root of the in-plane area of the finite element. By expressing the cohesive law as $t^{cr}=g\left(\omega^{cr}\right)$ and combining Equations (4) and (5), system of non-linear equations is obtained that can be solved for the cracking strain using an iterative method:

(6) $$\begin{array}{cc} R^{T} & \cdot \left[D_{e}:\varepsilon -D_{e}:\left(R\cdot \varepsilon_{c}^{cr}\cdot R^{T}\right)\right]\cdot n_{2}\\ \; & =g\left\{\left[\left(2\varepsilon_{c}^{cr}:n_{1}^{cr}⊗n_{2}^{cr}\right)n_{1}^{cr}+\left(\varepsilon_{c}^{cr}:n_{2}^{cr}⊗n_{2}^{cr}\right)n_{2}^{cr}+\left(2\varepsilon_{c}^{cr}:n_{2}^{cr}⊗n_{3}^{cr}\right)n_{3}^{cr}\right]l^{*}\right\}. \end{array}$$

Camanho et al. [38] proposed a modified version of the constitutive model, originally proposed by Turon et al. [42], based on a linear softening cohesive law in the context of a SCM. This cohesive law defines the tractions acting on the fracture plane as follows [34]:

(7) $$t^{cr}=t_{i}^{cr}\left(d_{SCM}\right)=\left[\begin{array}{c} \left(1-d_{SCM}\right)\frac{\left|\omega_{1}^{cr}\right|}{\omega_{f}^{m}}{\bar{t}}_{1}^{cr}\\ \left(1-d_{SCM}\right)\frac{\left|\omega_{2}^{cr}\right|}{\omega_{f}^{m}}{\bar{t}}_{2}^{cr}\\ \left(1-d_{SCM}\right)\frac{\left|\omega_{3}^{cr}\right|}{\omega_{f}^{m}}{\bar{t}}_{3}^{cr} \end{array}\right]+\frac{\langle -\omega_{2}^{cr}\rangle }{\left|\omega_{2}^{cr}\right|}\left[\begin{array}{c} d_{SCM}\;\mu_{L}\frac{\omega_{1}^{cr}}{\omega_{f}^{m}}\left|t_{2}^{cr}\right|\\ t_{2}^{cr}-\left(1-d_{SCM}\right)\frac{\left|\omega_{2}^{cr}\right|}{\omega_{f}^{m}}\;{\bar{t}}_{2}^{cr}\\ d_{SCM}\;\mu_{T}\frac{\omega_{3}^{cr}}{\omega_{f}^{m}}\left|t_{2}^{cr}\right| \end{array}\right],$$

where $d_{SCM}$ is a scalar damage variable, ${\bar{t}}_{i}^{cr}$ are the scalar components of the traction tensor on the failure plane at the onset of transverse failure, $\omega_{i}^{cr}$ are the scalar components of the displacement jump vector, $\omega_{f}^{m}$ is the equivalent displacement jump at failure under mixed-mode loading conditions, $\varepsilon_{22}^{cr}$ and $\varepsilon_{c_{22}}^{cr}$ are the scalar components of the total and cracking strain tensors, respectively, $\mu_{L}$ and $\mu_{T}$ are the frictional coefficients in the longitudinal and transverse directions, respectively, and 〈·〉 are the Macauley brackets defined as $\langle x\rangle =\frac{1}{2}\left(x+\left|x\right|\right)$. Here, it is assumed that the frictional coefficients in the longitudinal and transverse directions are equal ($\mu_{L}=\mu_{T}=\mu$).

To obtain the damage variable for the SCM ($d_{SCM}$), a suitable loading function L, as proposed in Ref. [42], is used:

(8) $$d_{SCM}=\max\left\{\;0,\;\max\left[L(\omega^{cr})\right]\right\},$$

with

(9) $$L\left(\omega^{cr}\right)=\min\left\{\frac{\lambda }{\omega_{f}^{m}},\;1\right\},$$

where λ is the equivalent displacement jump defined as follows [38]:

(10) $$\lambda =\sqrt{{\left(\omega_{1}^{cr}\right)}^{2}+{\left(\omega_{3}^{cr}\right)}^{2}+{\langle \omega_{2}^{cr}\rangle }^{2}}.$$

The equivalent displacement jump at failure, $\omega_{f}^{m}$, is defined using the BK criterion for crack propagation under mixed-mode loading conditions [38]:

(11) $$\omega_{f}^{m}=\frac{2\left(G_{\mathrm{Ic}}+AB^{\eta }\right)}{{\bar{t}}^{cr}},$$

where $A=G_{\mathrm{IIc}}-G_{\mathrm{Ic}}$, *B* is the mode ratio, η is the mixed-mode interaction parameter used in the BK law, $G_{\mathrm{Ic}}$ and $G_{\mathrm{IIc}}$ are mode I and mode II intralaminar fracture toughness associated with transverse failure mechanisms, respectively, and ${\bar{t}}^{cr}$ is the norm of the traction tensor at the onset of transverse failure:

(12) $${\bar{t}}^{cr}=\sqrt{{\left(\;{\bar{t}}_{1}^{cr}\;\right)}^{2}+{\left(\;{\bar{t}}_{3}^{cr}\;\right)}^{2}+{\langle \;{\bar{t}}_{2}^{cr}\;\rangle }^{2}}.$$

The scheme of the numerical solution of Equation (6) for the cracking strain using the Newton–Raphson method is presented in Ref. [38]. The residual for this system of non-linear equations is provided as follows:

(13) $$r\left(\varepsilon_{c}^{cr}\right)=f\left(\varepsilon_{c}^{cr}\right)-g\left(\varepsilon_{c}^{cr}\right),$$

where $f(\varepsilon_{c}^{cr})$ and $g(\varepsilon_{c}^{cr})$ are the left-hand side and right-hand side of Equation (6), respectively. After reaching the convergence of the iterative method, with the obtained cracking strains, the stress tensor is easily updated using Equation (3).

### 2.2. Development of a Bi-Linear Softening Law Based on a 3D Kink Band Model

Following the definition of the analytical framework related to transverse matrix cracking, the onset of fiber-dominated failure was predicted using the 3D invariant-based failure theory, as illustrated in the flowchart of the implemented model (Figure 2). Initiation of fiber failure activates the longitudinal damage variable $d_{1}$, which is calculated within the framework of CDM.

**Figure 4:**
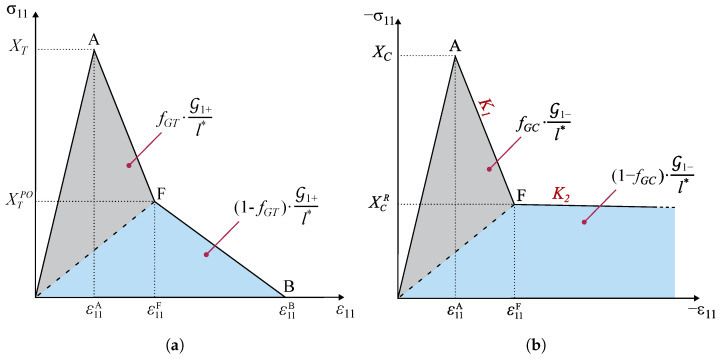
Uniaxial response and softening law under longitudinal tension (**a**) and longitudinal compression (**b**).

The damage propagation functions for longitudinal failure mechanisms must account for the different energy-dissipating mechanisms associated with the propagation of a crack perpendicularly to the reinforcing fibers, which include fiber and matrix fractures and fiber–matrix debonding [38]. For this reason, Camanho et al. [38] suggested the use of bi-linear softening laws [30,31]. Bi-linear softening (Figure 4) is defined by the maximum stress, i.e., the longitudinal strengths ($X_{T}$ and $X_{C}$), by the stress corresponding to the modification of the softening slope (the pull-out stress $X_{T}^{PO}$ = $f_{XT}X_{T}$ in tension and the residual stress plateau $X_{C}^{R}$ = $f_{XC}X_{C}$ in compression), and by the partition of the dissipated energy per unit volume associated with each linear softening regime [34]. The idea behind the formulation of the cited model relies on the fulfilment of the second law of thermodynamics and, thus, preventing physically inadmissible damage evolution without energy dissipation.

The characteristic length, $l^{*}$, introduced for the SCM, was also used for this model to ensure that the numerical solution is independent of mesh refinement, recalling Bazant’s crack band model [32]. The implemented model accounts for this characteristic length using a normalized value of the fracture toughness, which is the energy dissipated per unit volume $g_{M}$, mentioned before and defined as follows:

$$g_{M}=\frac{G_{M}}{l^{*}},$$

where $G_{M}$ is the fracture toughness, and *M* allows us to identify the two longitudinal damage laws: under tension (M=1+) or compression (M=1−) [31].

The full details of the theoretical background and the implementation of the damage model for longitudinal failure mechanisms are provided in Refs. [30,31].

Following this damage model, Zhuang et al. [34,39] proposed a modification to enhance the accuracy of failure predictions using a fully 3D failure theory, such as the 3D invariant-based failure theory. However, as mentioned above, the theories defining the limit of the elastic region and the ones for the bi-linear softening law were coherent for the tensile case, based on the maximum strain criterion, but not in compression, where the 3D kinking model was used only for failure onset and the maximum stress criterion for damage evolution. This simplification adopted by Zhuang et al. [34,39] aimed to facilitate mesomodel implementation by employing similar damage evolution laws for longitudinal tensile and compressive failure and to avoid the numerical computation of additional parameters of the damage evolution law for longitudinal compressive failure mechanisms. However, this simplification suffered from the following disadvantages:

- The damage threshold variable (failure index) used for the onset of longitudinal compressive failure mechanisms may be significantly different from the one used in the propagation phase;
- Even though the effect of the stress components other than the longitudinal stress is taken into account for the onset of fiber kinking, it is not taken into account in the propagation of the kink band (this may not represent the actual physics of kink band propagation on complex, compression-dominated stress states).

To overcome these limitations in the description of kink band formation and broadening, the 3D invariant-based fiber kinking model was implemented into the corresponding damage evolution law. Therefore, for the first time, the full set of 3D invariant-based failure criteria was implemented for the onset and propagation of intralaminar damage within a meso-scale damage model.

Following the experimental findings of Moran et al. [43], the damage model for longitudinal compressive failure predicts the formation of a kink band, followed by linear softening until reaching a plateau, corresponding to kink band broadening at constant stress.

For a complete definition of the constitutive model, it is necessary to introduce the internal variables and the relation between the the damage variables. The internal variables $r_{M}$ represent the elastic domain thresholds so the level of elastic strains can be achieved before the accumulation of additional damage, and they are also related to the damage state of each variable by the damage evolution laws [31]. When the material is undamaged, before damage onset, the value of $r_{M}$ is lower than 1, and the damage variable is still equal to zero $d_{M}\left(r_{M}<1\right)=0$. In this model, the internal variables are implemented using the 3D invariant-based failure criteria for predicting the full post-peak response of the material.

For the definition of the bi-linear softening formulation for kink band broadening, it is required to separately consider the numerical description of each linear branch of the damage evolution law. The first branch can be characterized by the maximum stress and the stress at the inflection point (point F from Figure 4b), $X_{C}$ and $X_{C}^{R}$, respectively, and by the first portion of energy dissipated per unit area, $G_{XC}$. The portions of mode-I longitudinal compressive fracture energy in a kink band ($G_{1-}$) are given as follows:

(14) $$\begin{array}{c} G_{XC}=f_{GC}\;G_{1-},\\ G_{X_{C}^{R}}=\left(1-f_{GC}\right)G_{1-}, \end{array}$$

where $f_{GC}$ is the fraction of the mode-I longitudinal compressive fracture energy corresponding to the initial slope of the respective damage law, $G_{XC}$ is the fracture energy for the first branch of the softening law, and $G_{X_{C}^{R}}$ is the fracture energy for the second branch [2]. It should be noted that, to achieve a damage evolution law, as shown in Figure 4b, $G_{1-}$ in Equation (14) is given a large number, and $f_{GC}$ is defined such that $G_{XC}$ equals the steady-state value of the intralaminar fracture toughness for longitudinal compressive failure [2].

In this implementation, the longitudinal component of the stress tensor at the intersection with the failure surface is used, which is denoted as $X_{C}^{*}$. The location of the intersection of the elastic stress vector with the failure surface is determined using an iterative scheme based on the Newton–Raphson method. This allows us to compute the exact longitudinal stress ($\sigma_{11}$) triggering the onset of fiber kinking, which, for instance, coincides with $X_{C}$ when the other stress components are negligible. Hence, this value will be the “effective” maximum stress of the first branch of the bi-linear softening law.

The value of the strain at the deflection point F can be easily determined by considering the corresponding area of dissipated energy:

(15) $$\varepsilon_{11}^{F}=-\frac{2\;G_{XC}E_{1}+l^{*}X_{C}^{*}X_{C}^{R}}{l^{*}E_{1}\left|X_{C}^{*}\right|}.$$

Since the previous parameters are given from the material properties and the longitudinal stress component at failure, the characterization of the first branch of the bi-linear softening law requires only an additional ingredient: its slope. Therefore, the slope of the first softening branch is calculated as follows:

(16) $$K_{1}=\frac{|X_{C}^{*}|-|X_{C}^{R}|}{|\varepsilon_{11}^{F}|-|\varepsilon_{11}^{A}|}=\frac{l^{*}X_{C}^{*}E_{1}\left(X_{C}^{*}-X_{C}^{R}\right)}{2\;G_{XC}E_{1}-l^{*}X_{C}^{*}\left(X_{C}^{*}-X_{C}^{R}\right)}.$$

Using a similar procedure, the second slope can be easily obtained as follows:

(17) $$K_{2}=\frac{0-|X_{C}^{R}|}{|\varepsilon_{11}^{B}|-|\varepsilon_{11}^{F}|}=\frac{l^{*}{\left(X_{C}^{R}\right)}^{2}E_{1}\left(1-d_{F1-}\right)}{2\;G_{X_{C}^{R}}E_{1}\left(1-d_{F1-}\right)-l^{*}{\left(X_{C}^{R}\right)}^{2}},$$

where $d_{F1-}$ is the damage variable at inflection point F, which is defined as a function of material properties only. It can be noted that the slope of the first branch $K_{1}$ can also be expressed as a function of $d_{F1-}$:

(18) $$K_{1}=\frac{|X_{C}^{*}|-|X_{C}^{R}|}{|\varepsilon_{11}^{F}|-|\varepsilon_{11}^{A}|}=\frac{|X_{C}^{*}\left|-\right|X_{C}^{R}|}{\frac{|X_{C}^{R}|}{\left(1-d_{F1-}\right)E_{1}}-\frac{|X_{C}^{*}|}{E_{1}}}.$$

The determination of $d_{F1-}$ is obtained combining Equations (16) and (18), which is given as follows:

(19) $$d_{F1-}=\frac{2\;G_{XC}E_{1}}{2\;G_{XC}\;E_{1}-l^{*}X_{C}^{*}X_{C}^{R}}.$$

Simple geometrical considerations allow the determination of the strain at final failure (point B):

(20) $$\varepsilon_{11}^{B}=-\frac{2\;G_{X_{C}^{R}}}{l^{*}\left|X_{C}^{R}\right|}.$$

It should noted that, for relatively large finite elements, the fulfillment of energy dissipation leads to a snap-back in the constitutive response. To avoid this, a critical finite element size is defined [31], which leads to the verification of the following condition:

(21) $$l^{*}\leq \frac{2E_{1}G_{1-}}{{\left(X_{C}^{*}\right)}^{2}}.$$

**Figure 5:**
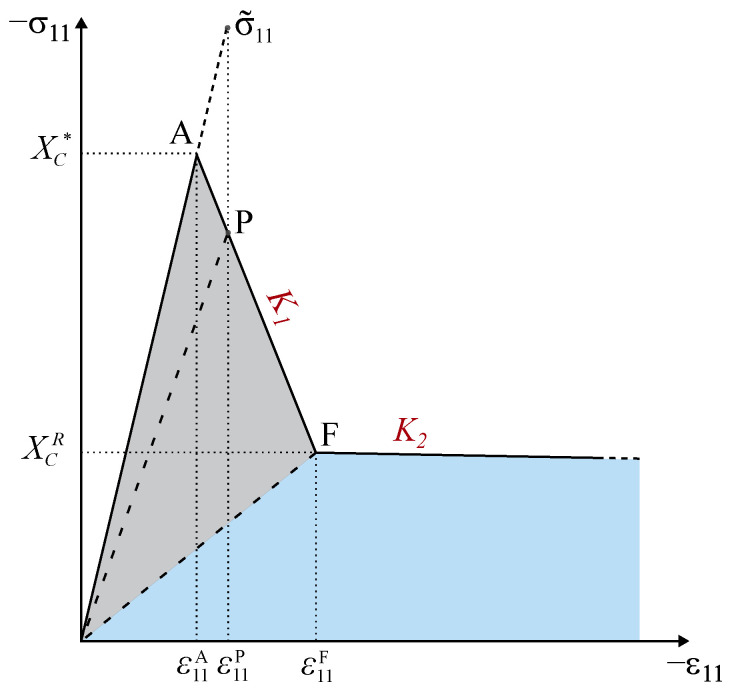
Schematic representation of the derivation of the first branch of a bi-linear damage evolution law.

Finally, for the formulation of the first branch of the bi-linear softening law, a generic point P (Figure 5) can be considered. The stress at P is given as follows:

(22) $$\sigma_{11}^{P}=\left|X_{C}^{*}\right|-K_{1}\left(|\varepsilon_{11}^{P}|-\frac{|X_{C}^{*}|}{E_{1}}\right).$$

### 2.3. Constitutive Response of Cohesive Elements

**Figure 6:**
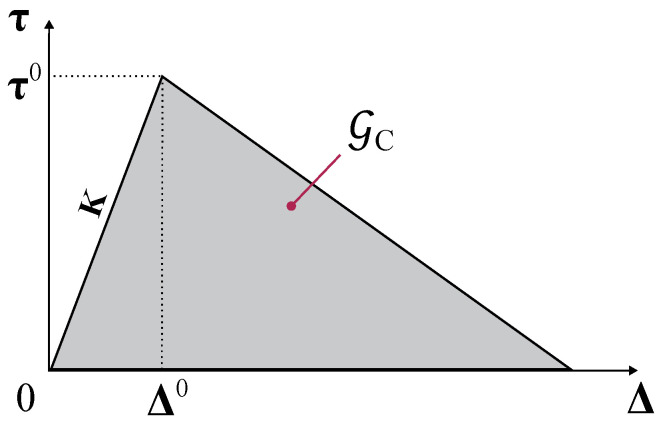
Linear traction separation response of cohesive elements.

The constitutive response of the cohesive elements is defined using a traction–separation law, which relates cohesive surface tractions, τ, to displacement jumps, Δ. A typical traction–separation response is shown in Figure 6, where *K* is the slope of the first linear regime representing the interface stiffness, $G_{c}$ is the fracture toughness, i.e., the area under the traction-displacement jump curve, and $\tau^{0}$ is the interfacial strength. In the present work, the FE solver used to simulate the mechanical behavior of composite materials is ABAQUS^®^ 2020, and finite-thickness COH3D8 cohesive elements were selected to model delamination. The available traction–separation model in ABAQUS^®^ 2020 assumes initially linear elastic behavior followed by the initiation and evolution of damage [37].

The criterion to predict the onset of delamination is the quadratic nominal stress criterion (ABAQUS^®^ Quads Damage), which is given as follows:

(23) $${\left(\frac{\langle \tau_{3}\rangle }{\tau_{3}^{0}}\right)}^{2}+{\left(\frac{\tau_{1}}{\tau_{1}^{0}}\right)}^{2}+{\left(\frac{\tau_{2}}{\tau_{2}^{0}}\right)}^{2}=1,$$

where the Macaulay bracket, 〈 〉, is used to specify that the compressive stress does not contribute to damage initiation, while $\tau_{3}$ is the traction stress acting in the normal through-thickness direction, $\tau_{1}$ and $\tau_{2}$ are the shear stresses, and $\tau_{1}^{0}$, $\tau_{2}^{0}$, and $\tau_{3}^{0}$ are the corresponding strengths.

Once delamination has been initiated, it is controlled by a propagation criterion based on the fracture energy (or critical energy release rate), which is specified as a material property. As shown in Figure 6, the material response is supposed to degrade according to a linear softening law, and the total crack opening takes place when the fracture toughness is completely dissipated. The variation of fracture toughness as a function of mode ratio in epoxy composites is calculated using the Benzeggagh and Kenane (BK) criterion extended to the 3D case [44]. The BK fracture criterion is particularly useful when the critical fracture energies during deformation along the first and the second shear directions are the same. It is given as follows:

(24) $$G_{\mathrm{Ic}}+\left(G_{\mathrm{IIc}}-G_{\mathrm{Ic}}\right){\left(\frac{G_{\mathrm{II}}+G_{\mathrm{III}}}{G_{I}+G_{\mathrm{II}}+G_{\mathrm{III}}}\right)}^{\eta }=G_{c},$$

where η is an experimentally derived parameter, $G_{\mathrm{Ic}}$ and $G_{\mathrm{IIc}}$ are mode I and II fracture toughness, and $G_{I}$, $G_{\mathrm{II}}$, and $G_{\mathrm{III}}$ are the energy release rates corresponding to fracture modes I, II, and III, and their sum is the total energy release rate.

For linear softening, ABAQUS^®^ uses an evolution of the damage variable, $D_{k}$, which enables us to track the state of an interface element (from 0, the undamaged state, to 1, fully damaged), which was proposed by Camanho and Dávila [45]:

(25) $$D_{k}=\frac{\Delta^{f}\left(\Delta -\Delta^{0}\right)}{\Delta \left(\Delta^{f}-\Delta^{0}\right)},$$

where Δ is the maximum relative displacement at a given increment, $\Delta^{f}$ is the displacement at failure, and $\Delta^{0}$ is the displacement at the onset of delamination.

## 3. Material Model Verification

A preliminary verification study of the meso-scale damage model was first performed at the single element level to check its correct numerical implementation. Verification was performed to assess the capability of the material model in capturing the onset and evolution of fiber and matrix-dominated failure. However, as the novelty of the proposed damage model refers to fiber-kinking, this section mainly presents the verification of the CDM model and the SCM model.

Firstly, single elements under longitudinal tension and compression were tested and subjected to uniaxial loading involving longitudinal damage progression and fiber-dominated failure. These simple stress states were imposed by means of null displacement and velocity boundary conditions applied to the surfaces of the element. The material selected for the present verification studies was IM7/8552 carbon-epoxy UD tape. The properties of this material are reported in Table 2, where $E_{1}$, $E_{2}$, and $E_{3}$ are the Young’s moduli, $G_{12}$, $G_{23}$, and $G_{13}$ are the shear moduli, and $\nu_{12}$, $\nu_{23}$, and $\nu_{13}$ are the Poisson’s ratios and ρ is the material density. $S_{T}$ and $S_{L}$ are, respectively, the transverse and in-plane shear strengths, $X_{C}$ is the longitudinal compressive strength, $X_{T}$ is the longitudinal tensile strength, $Y_{C}$ and $Y_{BC}$ are, respectively, the transverse uniaxial and biaxial compressive strengths, and $Y_{T}$ and $Y_{BT}$ are, respectively, the transverse uniaxial and biaxial tensile strengths. $G_{\mathrm{Ic}}$ and $G_{\mathrm{IIc}}$ are, respectively, the fracture toughness under pure mode I and under pure mode II loading, $G_{1-}$ and $G_{1+}$ are, respectively, the steady state propagation values of the fracture toughness for tensile fracture propagation and for kink band formation, η is the mixed-mode interaction parameter used in the BK law, and μ is the frictional coefficient implemented in the SCM.

**Figure 7:**
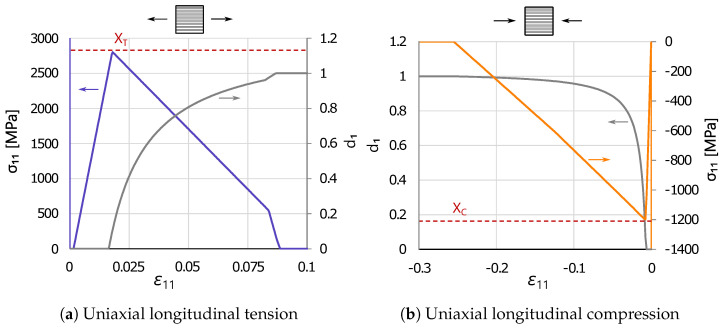
Verification study at the single element level showing the longitudinal damage variable ($d_{1}$, in grey) and longitudinal stress ($\sigma_{11}$, in blue and orange) evolution under uniaxial longitudinal tension (**a**) and compression (**b**).

For these single-element tests, a cubic eight-node linear hexahedral finite element with reduced integration (C3D8R) was used. ABAQUS^®^/Explicit 2020 [37] was used to predict the mechanical behavior of the element up to collapse by means of the damage model implemented in a VUMAT subroutine, written in “FORTRAN 90”. The obtained results are shown in Figure 7, including the evolution of the longitudinal stress $\sigma_{11}$ and fiber-dominated damage variable $d_{1}$ with longitudinal strain $\varepsilon_{11}$ for a single element of dimensions 1 × 1 × 1 mm^3^ under longitudinal tension and compression.

It can be observed that the evolution of the longitudinal stress and damage variable is correctly captured up to collapse. In particular, the stress level at the onset of failure under uniaxial tension and compression is well predicted, and the evolution of the damage variable $d_{1}$ follows the expected curve. The change in softening slope can be appreciated mainly in the tensile case, as the portion of dissipated energy in the first branch $\left(f_{GT}\cdot G_{1+}\right)$ is assumed to be significantly higher than the one in the second branch $\left(\left(1-f_{GT}\right)\cdot G_{1+}\right)$. In fact, the bi-linear softening law under compression is characterized by two branches with a similar slope (i.e., $K_{1}$ is similar to $K_{2}$, from Figure 5), as well as the two portions of dissipated fracture energy.

Then, a more complex test was run to verify the material response of IM7/8552 under an increasing level of longitudinal compression and constant biaxial transverse pressure. This verification study was performed on a single C3D8R element of dimensions 0.1 × 0.1 × 0.1 mm^3^.

**Figure 8:**
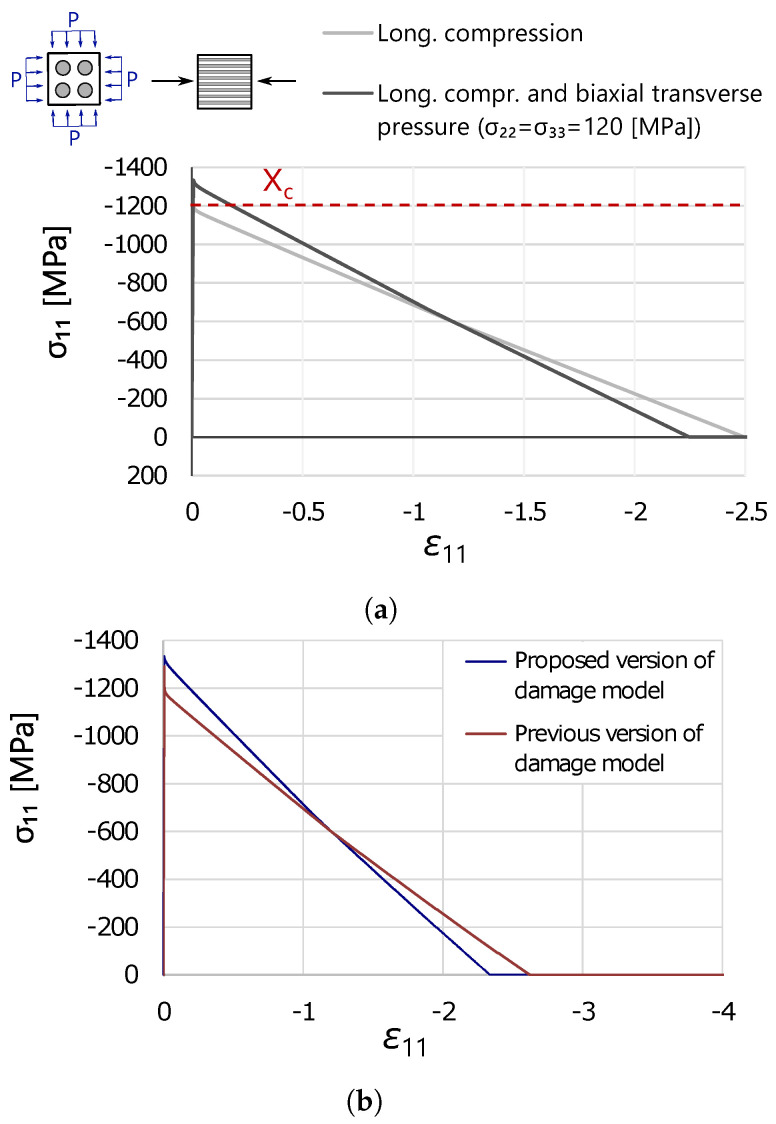
Verification study at the single element level under triaxial loading, realized by applying biaxial transverse pressure ($\sigma_{22}=\sigma_{33}=$ 120.0 MPa) and longitudinal compression: (**a**) comparison between the triaxial case and the uniaxial case; (**b**) comparison between the proposed and the previous versions of the longitudinal degradation models under triaxial compression.

The results of this test are shown in Figure 8. In particular, in Figure 8a, the results obtained for this test are compared to the ones under uniaxial longitudinal compression in order to highlight the effect of the applied biaxial transverse pressure. Figure 8b provides a comparison between the results obtained with the proposed damage model and its previous version.

It can be observed in Figure 8a that the implemented damage model is able to capture the increase in longitudinal compressive strength due to hydrostatic pressure by means of the implemented 3D invariant-based failure criteria as damage activation functions. Furthermore, softening curves are well approximated until final collapse, which occurs at two different longitudinal strains, confirming that the energy dissipated due to degradation is the same. The exact values of strain at failure (calculated using Equation (20)) are verified by means of a numerical script. Finally, Figure 8b shows how the previous model was also able to capture the onset of fiber-kinking in the presence of hydrostatic pressure, but the softening curve did not account for this effect, dropping suddenly to $X_{C}$. Hence, the previous version of the CDM model could not predict the effect of hydrostatic pressure on the kink band formation and broadening.

Additional tests were performed for different material systems and element sizes to verify the robustness of the model. The results of these tests confirmed the correct implementation of the numerical model, and the expected mechanical response in the fiber direction of FRP was captured for all cases.

Following the verification tests on the CDM model for damage propagation in the longitudinal direction, verification of the implementation of the SCM was also performed to assess the evolution of matrix-dominated failure, as described in Section 2.1.

With this aim, single C3D8R elements were subjected to uniaxial and simple shear stress states to trigger matrix cracking onset and propagation. In particular, five tests were performed by applying the following loading conditions: transverse tension, transverse compression, in-plane shear, out-of-plane shear, and transverse shear. The material used for these studies was IM7/8552 UD tape, and its properties can be found in Table 2. For all tests, the element dimensions were 0.1 × 0.1 × 0.1 mm^3^.

**Figure 9:**
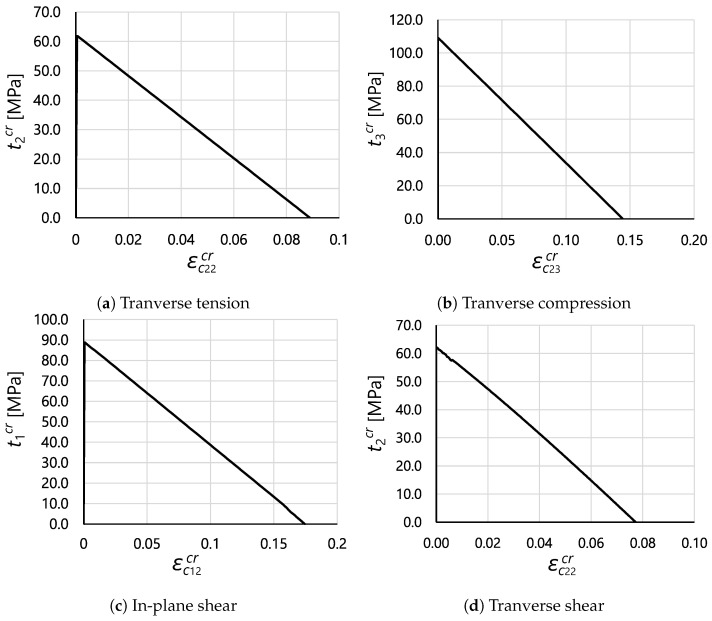
Numerical results of single element test under different loading conditions for matrix-dominated failure.

The obtained results are shown in Figure 9, where traction–cracking strain curves are provided for each test. In all cases, the model was able to correctly predict the onset of transverse damage mechanisms, as well as the computed fracture energy up to final failure. The latter was verified by comparing the area below the traction–cracking strain relations with the expected dissipated energy. For each of the cases, the predicted fracture angle α was also correctly computed.

## 4. Materials and Methods

### 4.1. Scaling Effects in Un-Notched Laminates

Table 3 summarizes the geometry of the multidirectional laminates tested to study the UNT strength of scaled specimens [4,5]. The material properties of IM7/8552 UD tape, as presented in Table 2, were used in this work. Additionally, in this study, it was important to account for in-situ strengths, as they can remarkably affect the mechanical response of laminates, especially when addressing the effect of ply-thickness scaling. Indeed, for the case of m = 8, the effective thickness of the inner 0° ply was 2 mm, and the corresponding in-situ strengths were significantly lower than the in-situ strengths of the inner single plies. These properties were derived using the approach proposed by Camanho et al. [14,35], and the obtained in-situ strengths for IM7/8552 are presented in Table 4.

### 4.2. Finite Element Modeling

The FE models were built using C3D8R solid elements for the prediction of the ply response. Furthermore, for each test case, a study of the influence of delamination in the evolution of damage up to collapse was performed, generating an additional model with interlaminar layers of COH3D8 cohesive elements between plies with different orientations. Therefore, following Arteiro et al. [49], multidirectional laminates were discretized using one element through the effective thickness of each ply block, defined as the sum of the thickness of the consecutive plies with the same orientation. This effective ply-by-ply discretization allowed us assigning, for each ply block, the correct in-situ strengths (Table 4), calculated as a function of the position and thickness of the ply block by means of different material cards.

**Figure 10:**
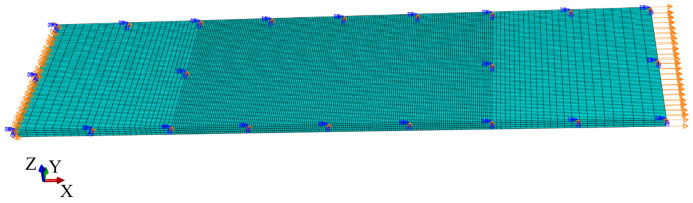
Mesh and boundary conditions for the simulation of a UNT specimen under tension, with ply-level scaling and m = 2: [45_2_/90_2_/−45_2_/0_2_]_s_.

Following Ref. [34], a multi-region strategy was employed for an accurate definition of in-plane FE sizes to minimize the computational cost of this numerical study. The laminates were divided into three regions (see Figure 10): a central region with a constant element size, lower than the critical value $l^{*}$, where material degradation was included, and two side regions, with biased mesh size growing from the centre to the edge, where the material response was kept linear-elastic for the entire simulation. The element size in the refined region was selected to be lower than the minimum critical value in both longitudinal and transverse directions (equal to 0.19 mm, calculated using Equation (21)), considering the respective in-situ strengths and fracture properties.

As mentioned above, delamination was predicted using COH3D8 cohesive elements, available in ABAQUS^®^/Explicit, with the same discretization used for modeling the intralaminar mechanical behavior and a thickness of 0.01 mm. Their constitutive response was defined using a traction–separation law, as described in Section 2.3. The interlaminar material properties of IM7/8552 were assumed to be equal to the corresponding values of the matrix-dominated intralaminar damage, given the similarity in the fracture modes. These parameters are presented in Table 5.

The numerical study was performed using ABAQUS^®^/Explicit with a VUMAT user subroutine to run the meso-scale damage model. The applied loading was simulated using a suitable velocity boundary condition introduced using a smooth step amplitude on the upper end of the laminate while clamping the lower end. The symmetry with respect to the laminate midplane was exploited by setting symmetry boundary conditions. As an example, the overall imposed boundary conditions for the ply-level scaled UNT specimen, with m = 2, under tensile loading are shown in Figure 10.

To suppress spurious energy modes (such as zero-energy mode), enhanced hourglass and distortion control (length ratio equal to 0.80) were employed in the elements where intralaminar damage was simulated. Furthermore, an efficient strategy to delete excessively distorted elements was implemented based on the element volume change, which was tracked by the determinant of the deformation gradient tensor $\det F=V/V_{0}$. Therefore, with the aim of imposing limits on large changes in element volume and allowing reliable results, the following conditions were specified for the deletion of intralaminar elements [50,51]:

(26) $$\mathrm{Delete}\;\mathrm{element}\;\mathrm{if}:\left\{\begin{array}{c} \;d_{1}>0.999\\ \;d_{SCM}>0.999\\ \;0<\det F<0.8\;\;\mathrm{or}\;\;\det F>1.2. \end{array}\right.$$

This simulation strategy prevents premature job termination due to excessive element distortion before final structure failure. When cohesive elements were included in the analysis, they were set to be deleted only when fully damaged, i.e., when their damage variable (Equation (25)) was equal to one.

## 5. Finite Element Predictions

**Figure 11:**
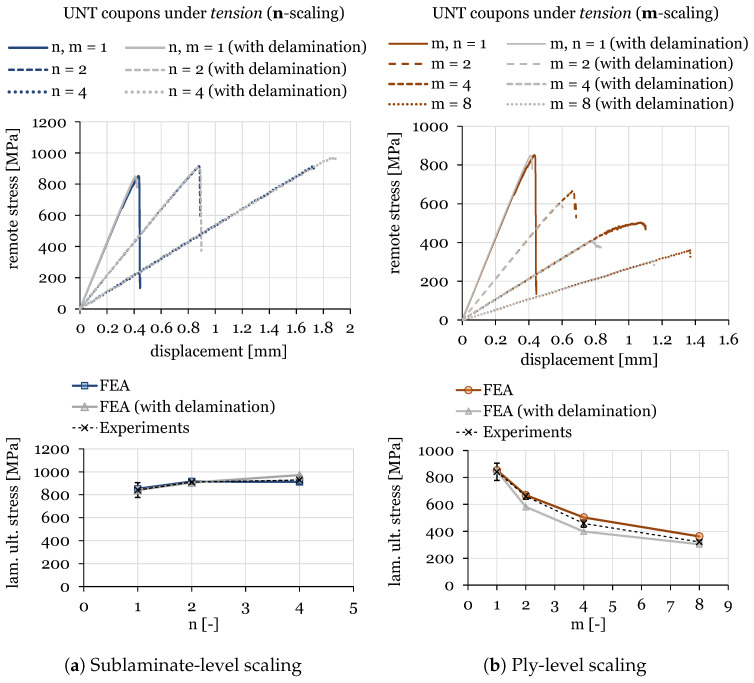
Size effect study on UNT quasi-isotropic ([45_m_/90_m_/−45_m_/0_m_]_ns_) specimens under tension with n-scaling (**a**) and m-scaling (**b**).

**Figure 12:**
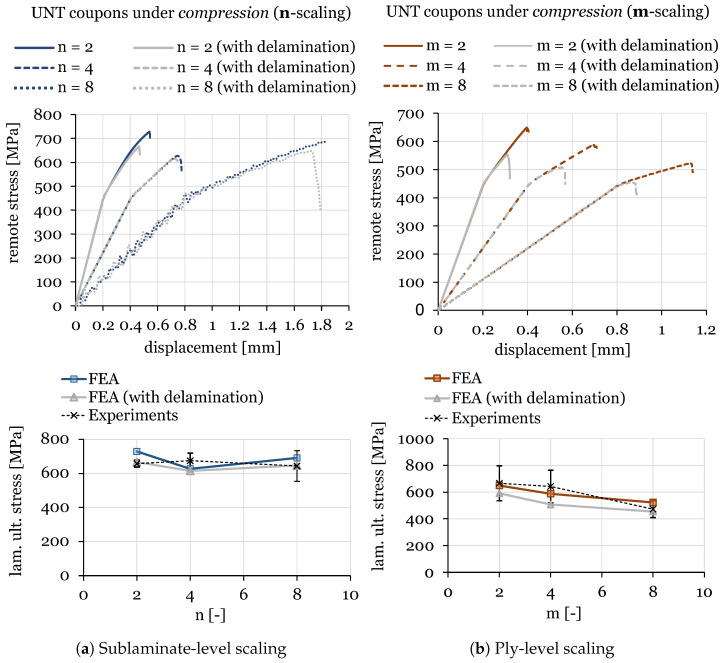
Size effect study on UNT quasi-isotropic ([45_m_/90_m_/−45_m_/0_m_]_ns_) specimens under compression with n-scaling (**a**) and m-scaling (**b**).

The numerical results for ply-level and sublaminate-level scaled tests on QI UNT specimens under tension and compression are shown in Figure 11 and Figure 12, respectively. A summary of the results of scaled tensile and compressive strengths, compared with experimental data and the associated standard deviation, is provided in Table 6.

It can be observed that the numerical predictions fit the experimental results quite well for ply-level and sublaminate-level scaled specimens for the two loading cases involving tension and compression. In fact, the scaling effect was well predicted by the proposed modeling strategies. In particular, relative errors between the mean strength measured experimentally and the one predicted numerically, including or neglecting the effect of delamination, are nearly always within the standard error and below 15%. Indeed, in a set of 12 cases, the only test where the predicted strength was above 15% error was the m = 4 specimen under compression, where a significant scatter was detected in the experiments, with a coefficient of variation (CV) of 19%.

Additionally, this work allowed us to challenge the proposed mesoscale model in the prediction of damage progression in thick multidirectional laminates with a nominal thickness of 8 mm. However, these cases are characterized by numerical oscillations that are an artefact of explicit dynamic modeling. This can be clearly observed in the case of n = 8 under compression. In general, a good agreement with experimental data was also achieved for thick UNT specimens.

Furthermore, it should be noted that delamination plays an important role in the prediction of UNT strengths, especially for cases with blocked plies (ply-level scaling). Indeed, the numerical models predicted an earlier failure driven by delamination due to the higher interlaminar stresses generated by thick inner ply blocks and lower in-situ strengths. In general, a modeling strategy with cohesive elements allows us to minimize the overprediction of UNT strength and, thus, it is required for this type of study.

**Figure 13:**
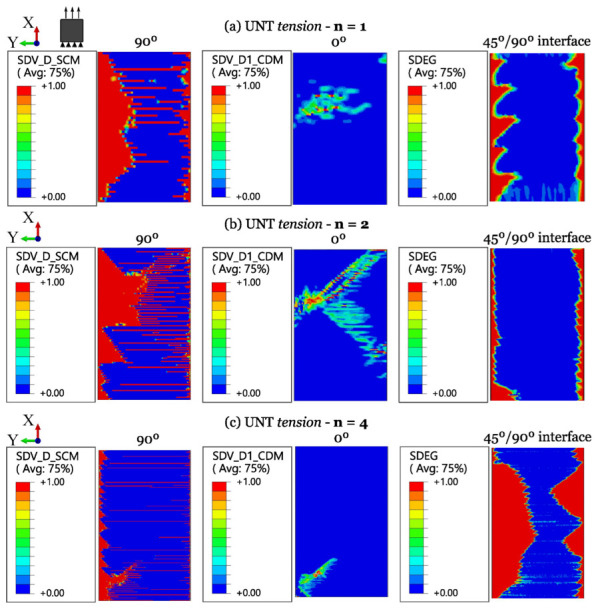
Damage progression in sublaminate-level scaled UNT specimens subjected to longitudinal tension.

The numerical predictions of damage progression in sublaminate-level scaled UNT specimens are shown in Figure 13. It includes the output fields showing the evolution of (i) matrix-dominated failure through the user-defined damage variable of the SCM (“SDV_D_SCM”), (ii) fiber-dominated failure by means of the user-defined damage variable of the CDM model (“SDV_D1_CDM”), and (iii) delamination using the ABAQUS^®^ internal damage variable (“SDEG”).

The simulations confirm that transverse matrix cracks are the first damage mechanism. Indeed, they start developing before reaching ultimate failure, providing less and less support to the 0° plies. Final collapse is predicted to be driven by delamination and fiber tensile failure after the large development of matrix cracks across the width of the specimens (see Figure 13). These predictions are in line with the experimental results, as delamination is found to have little influence on UNT tensile strengths. The increase in UNT strength with sublaminate-level scaling can be attributed to a lower extent of matrix cracking (“SDV_D_SCM”) with increasing laminate size.

**Figure 14:**
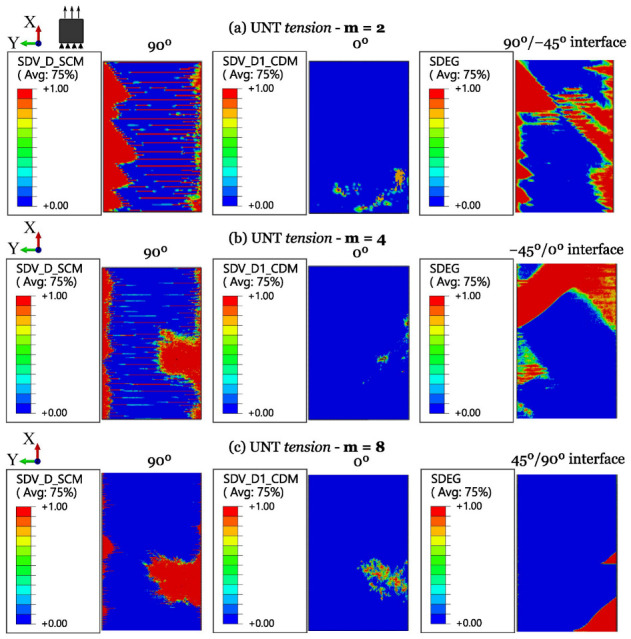
Damage progression in ply-level scaled UNT specimens subjected to longitudinal tension.

For m = 2 (Figure 14a), the predicted failure events are also in line with the experimental findings. Indeed, transverse matrix cracks develop slowly until delamination occurs at the interface between the 90° and −45° plies, which quickly leads to catastrophic failure. The predictions for m = 4 (Figure 14b) are also in agreement with the experiments since a large delamination occurred between the −45° and 0° plies, resulting in a drop in load. Finally, in the specimen with m = 8 (Figure 14c), transverse matrix cracks developed earlier, followed by delamination forming small triangular patches, starting from the matrix cracks at the free edge, as observed in the experiments [4]. The decrease in UNT strength with ply-level scaling can be attributed to an earlier occurrence of matrix cracking (“SDV_D_SCM”) and delamination (“SDEG”) with increasing laminate size.

**Figure 15:**
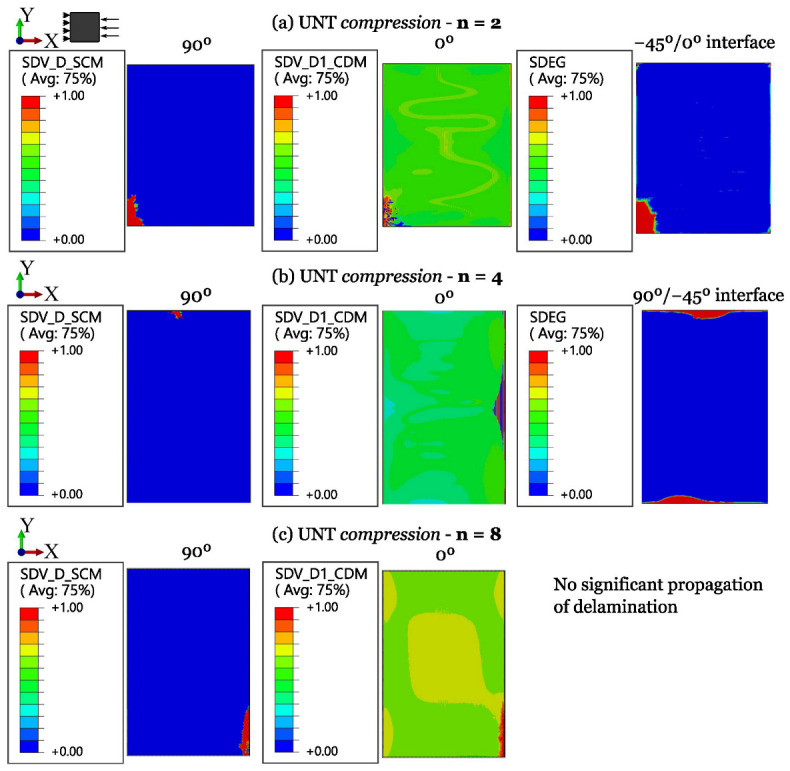
Damage progression in sublaminate-level scaled UNT specimens subjected to longitudinal compression.

**Figure 16:**
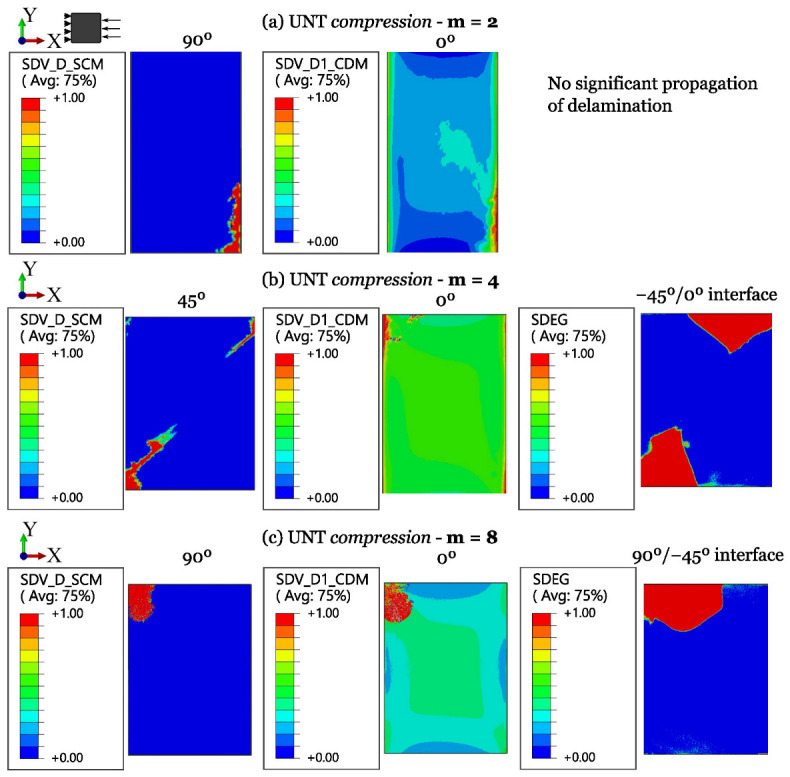
Damage progression in ply-level scaled UNT specimens subjected to longitudinal compression.

The numerical results on UNT compressive strength, shown in Figure 15 and Figure 16, highlight that the failure scenario is always dominated by fiber kinking, usually starting from the free edges. For ply-level scaled specimens (m-scaling, Figure 16), the influence of delamination is relevant in the progressive evolution of damage, contributing to earlier collapse. The issues found in the experiments result in a difficult correlation, but the overall response is in agreement with the experimental observations. Furthermore, since these failure events grow in a dynamic way, it results in a challenging capturing the full progression of damage and, often, only the initial stage is available for post-processing. However, in this work, a clear load drop was obtained for all the cases.

## 6. Conclusions

Following the extension of a previously developed model [34,38,39] to consistently capture complex 3D stress states in the prediction of both onset and broadening of longitudinal and transverse failure mechanisms, an extended verification study at the single element level was performed, proving the correct implementation of the model, and showing that detailed analysis can be correctly realized. Furthermore, the correct implementation of the effect of different levels of hydrostatic pressure on the expected apparent increase of compressive strength was verified with recourse to single finite element tests (Section 3).

Then, a validation study was performed considering experimental data at the coupon level. In particular, the proposed damage model was employed to predict the size effect in quasi-isotropic un-notched coupons under tensile and compressive loading. Two types of scaling were addressed: sublaminate-level scaling, by means of repeating the sublaminate stacking sequence, and ply-level scaling, realized by changing the effective thickness of each ply block. The predictions were in agreement with the experimental findings in terms of quantitative predictions (with an acceptable deviation from the mean value) and damage progression before final failure. Moreover, the developed model was able to capture the size effect in the un-notched strength well and highlighted the importance of properly capturing delamination in the prediction of scaling effects in composite laminates.

## Figures and Tables

**Table 1:** Determination of the fracture angle α, modeled using Refs. [34,35,39,41].

Conditions	Determination of Fracture Angle α
$\left\{\;I_{3}<0\;,\;\;\;\sigma_{22}<-|S_{T}|\;,\;\;\;|\sigma_{33}|>|\sigma_{22}|\;\right\}$ or $\left\{\;I_{3}<0\;,\;\;\;\sigma_{22}>-|S_{T}|\;,\;\;\;\sigma_{33}<-|S_{T}|\;\right\}$	$\alpha =\mathrm{sgn}\left\{\sigma_{23}\right\}\;\arcsin\sqrt{\frac{S_{T}}{\min\{|\sigma_{22}|,|Y_{C}|\}}}$
$\left\{\;I_{3}<0\;,\;\;\;\sigma_{22}<-|S_{T}|\;,\;\;\;|\sigma_{22}|>|\sigma_{33}|\;\right\}$ or $\{\;I_{3}<0\;,\;\;\;\left[\;\sigma_{22},\sigma_{33}>-\left|S_{T}\right|\;\right]\;,$ $\left[\;|\sigma_{23}\left|,\right|\sigma_{22}-\sigma_{33}\left|,\right|\sigma_{12}\left|,\right|\sigma_{13}|≊0\;\right]\;\}$	$\alpha =\mathrm{sgn}\left\{\sigma_{23}\right\}\;\arccos\sqrt{\frac{S_{T}}{\min\{|\sigma_{33}|,|Y_{C}|\}}}$
$\{\;\left[\;I_{3}<0\;,\;\;\;\sigma_{22},\sigma_{33}>-\left|S_{T}\right|\;\right]$ or $\left[\;I_{3}>0\;\right]$ and $|\alpha_{1}I_{1}+\alpha_{3}I_{3}+\alpha_{32}I_{3}\left|>\right|\alpha_{2}I_{2}\left|\;\right\}$	$\alpha =\arctan\left(\frac{\sigma_{13}}{\sigma_{12}}\right)$
$\{\;I_{3}<0$ and $\left[\;|\sigma_{23}\left|,\right|\sigma_{22}-\sigma_{33}\left|,\right|\sigma_{12}\left|,\right|\sigma_{13}|≊0\;\right]\}$	α=0
$\{\;\left[\;I_{3}<0\;,\;\;\;\sigma_{22},\sigma_{33}>-\left|S_{T}\right|\;\right]$ or $\left[\;I_{3}>0\;\right]$ and $|\alpha_{1}I_{1}+\alpha_{3}I_{3}+\alpha_{32}I_{3}\left|<\right|\alpha_{2}I_{2}\left|\;\right\}$	$\alpha =\frac{1}{2}\;\arctan\left(\frac{2\sigma_{23}}{\sigma_{22}-\sigma_{33}}\right)$

**Table 2:** Material properties of IM7/8552 UD tape used for verification of the implemented damage model.

Property	Value	Units	Reference/Equation
**Young’s moduli**			
$E_{1}$	171.42	GPa	[14]
$E_{2}=E_{3}$	9.08	GPa	[14]
**Poisson’s ratios**			
$\nu_{12}=\nu_{13}$	0.32	-	[14]
$\nu_{23}$	0.487	-	[14]
**Shear moduli**			
$G_{12}=G_{13}$	5.29	GPa	[14]
$G_{23}$	3.05	GPa	$G_{23}=\frac{E_{2}}{2+2\cdot \nu_{23}}$
**Density**			
ρ	1.59	g/cm^3^	[14]
**Longitudinal strengths**			
$X_{T}$	2806.0	MPa	[4]
$X_{C}$	−1200.1	MPa	[14]
$f_{XT}$	0.4	-	[2]
$f_{XC}$	0.506	-	Note (1)
**Transverse strengths**			
$Y_{T}$	62.3	MPa	[14]
$Y_{BT}$	38.7	MPa	[46]
$Y_{C}$	−253.7	MPa	[47]
$Y_{BC}$	−600.0	MPa	[46]
$S_{L}$	89.6	MPa	[48]
$S_{T}$	62.3	MPa	[14]
**Longitudinal fiber-dominated fracture**			
$G_{1-}$	150.0	kJ/m^2^	Note (1)
$G_{1+}$	134.7	kJ/m^2^	[38]
$f_{GC}$	0.484	-	Note (1)
$f_{GT}$	0.824	-	Note (2)
**Transverse matrix-dominated fracture**			
$G_{\mathrm{Ic}}$	0.277	kJ/m^2^	[14]
$G_{\mathrm{IIc}}$	0.788	kJ/m^2^	[14]
η	1.634	-	[38]
μ	0.4	-	[34]

(1) Inversely identified using one OHC simulation, following Ref. [2]. (2) Inversely identified using one OHT simulation, following Ref. [2].

**Table 3:** Dimensions and stacking sequence of scaled UNT quasi-isotropic laminates [4,5].

Specimen Type	Layup	Gauge Length × Width	Nominal Thickness
([45_m_/90_m_/−45_m_/0_m_]_ns_)		[mm × mm]	[mm]
**Tension** [4]			
(sublaminate-level scaling)			
n, m = 1	[45/90/−45/0]_s_	30 × 8	1.0
n = 2	[45/90/−45/0]_2s_	60 × 16	2.0
n = 4	[45/90/−45/0]_4s_	120 × 32	4.0
(ply-level scaling)			
m = 2	[45_2_/90_2_/−45_2_/0_2_]_s_	60 × 16	2.0
m = 4	[45_4_/90_4_/−45_4_/0_4_]_s_	120 × 32	4.0
m = 8	[45_8_/90_8_/−45_8_/0_8_]_s_	240 × 64	8.0
**Compression** [5]			
(sublaminate-level scaling)			
n = 2	[45/90/−45/0]_2s_	30 × 30	2.0
n = 4	[45/90/−45/0]_4s_	60 × 60	4.0
n = 8	[45/90/−45/0]_8s_	120 × 60	8.0
(ply-level scaling)			
m = 2	[45_2_/90_2_/−45_2_/0_2_]_s_	30 × 30	2.0
m = 4	[45_4_/90_4_/−45_4_/0_4_]_s_	60 × 60	4.0
m = 8	[45_8_/90_8_/−45_8_/0_8_]_s_	120 × 120	8.0

**Table 4:** In-situ properties of the IM7/8552 carbon–epoxy material system.

Ply Position (Thickness)	In-Situ Strengths [MPa]					
	$Y_{T}$	$Y_{\mathrm{BT}}$	$Y_{C}$	$Y_{\mathrm{BC}}$	$S_{L}$	$S_{T}$
Inner ($t_{\mathrm{ply}}$)	160.2	160.0	−437.6	−600.0	179.2	124.6
Inner ($2\cdot t_{\mathrm{ply}}$)	113.3	82.7	−364.0	−600.0	140.0	97.3
Inner ($4\cdot t_{\mathrm{ply}}$)	98.7	63.5	−297.6	−600.0	108.6	75.5
Inner ($8\cdot t_{\mathrm{ply}}$)	98.7	63.5	−297.6	−600.0	108.6	75.5
Inner ($16\cdot t_{\mathrm{ply}}$)	98.7	63.5	−297.6	−600.0	108.6	75.5
Outer ($t_{\mathrm{ply}}$)	100.8	64.3	−364.0	−600.0	140.0	97.3
Outer ($2\cdot t_{\mathrm{ply}}$)	71.3	43.1	−293.8	−600.0	106.9	74.3
Outer ($4\cdot t_{\mathrm{ply}}$)	62.3	38.7	−253.7	−600.0	89.6	62.3
Outer ($8\cdot t_{\mathrm{ply}}$)	62.3	38.7	−253.7	−600.0	89.6	62.3

**Table 5:** Interlaminar properties of the IM7/8552 carbon–epoxy material system.

Property	Value	Units	Reference
**Interface stiffness**			
*K*	1.0$\;\times \;10^{6}$	N/mm^3^	[2]
**Interface maximum strengths**			
$\tau_{1}^{0}=\tau_{2}^{0}$	89.6	MPa	[48]
$\tau_{3}^{0}$	62.3	MPa	[2]
**Interface critical energy release rate**			
$G_{\mathrm{Ic}}$	0.277	kJ/m^2^	[14]
$G_{\mathrm{IIc}}=G_{\mathrm{IIIc}}$	0.788	kJ/m^2^	[14]
**Mixed-mode interaction parameter**			
η	1.634	-	[38]

**Table 6:** Comparison between experimental results from Refs. [4,5] and the proposed mesoscale damage model on the UNT strength of scaled QI specimens.

Specimen Type	Experiments	CV	Predictions	Error	Predictions with	Error
([45_m_/90_m_/−45_m_/0_m_]_ns_)	[MPa]	[%]	[MPa]	[%]	Delam. [MPa]	[%]
**Tension**						
(sublaminate-level scaling)						
n, m = 1	842	±7.6	853	+1.3	841	−0.2
n = 2	911	±2.0	915	+0.4	906	−0.5
n = 4	929	±3.9	911	−1.9	971	+4.5
(ply-level scaling)						
m = 2	660	±3.3	669	+1.3	582	−11.8
m = 4	458	±5.8	503	+9.7	398	−13.1
m = 8	321	±2.9	362	+12.9	304	−5.3
**Compression**						
(sublaminate-level scaling)						
n = 2	658	±3.15	729	+10.8	667	+1.3
n = 4	675	±6.6	627	−7.1	615	−8.8
n = 8	644	±14.0	691	+7.2	647	+0.5
(ply-level scaling)						
m = 2	666	±19.6	649	−2.5	591	−11.3
m = 4	642	±19.0	587	−8.5	508	−20.9
m = 8	472	±13.4	522	+10.7	454	−3.7

## Data Availability

The original contributions presented in the study are included in the article, further inquiries can be directed to the corresponding authors.

## Abbreviations

The following abbreviations are used in this manuscript:

3D	Three-dimensional
BBA	Building Block Approach
CDM	Continuum Damage Mechanics
CFRP	Carbon-fiber-reinforced Polymer
FRP	Fiber-reinforced Polymer
NT	Notched
QI	Quasi-isotropic
SCM	Smeared Crack Model
UNT	Un-notched
